# Development of a 7-miRNA prognostic signature for patients with bladder cancer

**DOI:** 10.18632/aging.204447

**Published:** 2022-12-21

**Authors:** Yingjie Xv, Ming Qiu, Zhaojun Liu, Mingzhao Xiao, Fen Wang

**Affiliations:** 1 Department of Urology, The First Affiliated Hospital of Chongqing Medical University, Chongqing, Yuzhong 400016, China; 2 Department of Urology, The People’s Hospital of Dazu, Chongqing, Dazu 402360, China; 3 Department of Cardiology, The First Affiliated Hospital of Chongqing Medical University, Chongqing, Yuzhong 400016, China; 4 Department of Pathology, The People’s Hospital of Dazu, Chongqing, Dazu 402360, China

**Keywords:** bladder cancer, miRNAs, prognostic signature, risk score, nomogram

## Abstract

Background: Bladder carcinoma (BC) represents one of the most prevalent malignant cancers, while predicting its clinical outcomes using traditional indicators is difficult. This study aimed to develop a miRNA signature for the prognostic prediction of patients with BC.

Materials and Methods: MiRNAs that expressed differentially were identified between 413 BC and 19 non-tumor patients, whose prognostic values were evaluated using univariate and multivariate Cox regression analyses. The independent prognostic factors were screened out and were used to establish a signature. The risk score of the signature was calculated. Receiver operating characteristic (ROC) curves and Kaplan-Meier curves were used to verify the predictive performance of the miRNA signature and the risk score. A nomogram was constructed which integrated with the miRNA signature and clinical parameters. Experiments were performed.

Results: 7 prognosis related miRNAs were selected as independent risk factors, and a 7-miRNA signature was constructed, with an area under ROC (AUC) of 0.721. The 7-miRNA-signature based risk score acts as an independent prognostic factor, with satisfactory predictive performance (AUC = 0.744). Increased miR-337-3p expressions were detected in tumor samples and BC cell lines than in non-tumorigenic tissues and cell lines. Experiments suggested that miR-337-3p induces the proliferation, migration, and invasion of BC cells.

Conclusion: The constructed 7-miRNA signature is a promising biomarker for predicting the prognosis of patients with BC, and miR-337-3p may act as a candidate therapeutic target in BC treatments.

## INTRODUCTION

Bladder cancer (BC) is the ninth most common cancer worldwide, generating 165,000 deaths annually [1, 2]. There are two clinical subtypes of BC: non-muscle-invasive bladder cancer (NMIBC) and muscle-invasive bladder cancer (MIBC), in which NMIBC accounts for approximately 75% of newly diagnosed bladder cancer patients. Most BC patients have long-term survival after undergoing the transurethral resection of the bladder tumor (TURBT). However, 10–15% of NMIBC patients may progress to MIBC, for which the 5-year overall survival (OS) is often less than 50% [3, 4]. Tumor-node-metastasis (TNM) classification and pathological grade are used in clinical settings to predict patient prognosis. However, the predictive ability for individual clinical outcomes remains inadequate since these classification systems are indicators at a population level [5]. Therefore, novel prognostic biomarkers that reveal the mechanisms underlying tumorigenesis for personalized prognosis prediction and the development of effective treatment strategies are urgently needed.

MicroRNA (miRNA), a member of the small non-coding RNA family, is 21–23 nucleotides long that can interact with the 3′ non-coding region of the target mRNA and regulate translation of corresponding proteins [6, 7]. Recent evidence indicates that miRNA regulates various steps in carcinogenesis, including cancer initiation, progression, metastasis, and patient survival [8, 9]. Various researches have proved that miRNAs could act as prognostic markers in various cancers, through microarray or RNA-sequencing technologies [10, 11]. Several miRNA signatures have been established to determine the prognosis of BC patients beyond their clinicopathological features, with satisfactory predictive performance [12–14]. However, only a few *in vitro* experiments have been conducted to confirm the correlation between discovered prognostic miRNAs and tumorigenesis.

In this study, differentially expressed miRNAs between BC and non-tumor tissues were mined via The Cancer Genome Atlas (TCGA) database, and a novel 7-miRNA based signature was established based on the prognosis relative miRNAs. Quantitative reverse transcription-based polymerase chain reaction (RT-qPCR) was used to detect the differentiated miRNAs’ expressions in BC cell lines and clinical samples, followed by *in vitro* functional analysis. The 7-miRNA signature established in our study proved to be a novel biomarker for predicting the progression and OS in BC patients.

## MATERIALS AND METHODS

### Data collection and screening of differentially expressed miRNAs

The miRNA expression profiles and corresponding clinical data (age, sex, grade, and TNM classification) of 413 BC patients and 19 non-tumor patients were obtained from TCGA [15]. Patients with incomplete data or ambiguous overall survival were excluded. The “limma” package of the R language (version 4.0.2) was used to screen out the differentially expressed miRNAs (*p* values < 0.05 and |Log2FC| values >1), which were retained for further analysis [16].

### Construction of miRNA prognostic signature

The mined differentially expressed miRNAs were subjected to univariate Cox regression analysis to explore whether there were associations between them and patients’ OS, and statistically significant miRNAs (*p* value < 0.001) were processed by multivariable Cox regression analysis. MiRNAs with statistically significance (*p* values < 0.05) in the multivariable Cox regression analysis were regarded as independent prognostic indicators for patients’ survival and were utilized to construct a 7-miRNA signature.

The miRNAs’ risk scores of each patient were calculated on the basis of the miRNAs’ coefficients, and the median risk score acted as a boundary to categorize low- and high-risk BC patients. The differences between OS of the high- and low-risk groups were compared via Kaplan-Meier survival curve analysis and survival curves. The predictive ability of the 7-miRNA signature was assessed using the receiver operating characteristic (ROC) curve analysis generated by R packages “ggplot2” and “survivalROC.” The univariate and multivariate Cox regression analyses were used to evaluate the signature’s independent predictive value in BC patients’ survival.

### Establishment and evaluation of the nomogram

A nomogram was established to predict the 1, 3, and 5-year OS in BC patients, through integration with the clinicopathological characteristics and risk score, using the “rms” package of the R (version 4.0.1). The clinical significance and concordance between the predicted and observed patients were evaluated via plotted time-dependent ROC curves.

### Prediction of target genes and function enrichment analysis

Online analysis tools, miRDB (http://www.mirdb.org/miRDB/index.html), miRTarBase, and TargetScan (http://www.targetscan.org/), were employed to predict the mRNAs that are downstream targets of our signature miRNAs. Owing to the large number of genes mined along with the three algorithms that determined different target binding, the ‘Draw Venn Diagram’ (http://bioinformatics.psb.ugent.be/webtools/Venn/) tool was used to screen the overlapping mRNAs, which were subsequently analyzed via the “enrichplot” package in the R language to annotate GO and enrich the KEGG pathway. The protein-protein interaction (PPI) network analysis of the target genes was conducted via the String Database.

### Clinical sample collection

The clinical samples, including 13 BC tissues and their paired non-tumor tissues, were obtained from histopathologically diagnosed BC patients who underwent curative resection at the First Affiliated Hospital of Chongqing Medical University between May 2020 and May 2021. Prior to conducting the study, the institutional Review Board of the First Affiliated Hospital of Chongqing Medical University approved the study protocols, and the requirement for patients’ informed consent was waived.

### Cell culture

The BC cell lines (T24, 5637, UMUC3, TCCSUP, and RT4) and a non-tumorigenic bladder cell line (SV-HUC-1) were purchased from the American Type Culture Collection (ATCC, Manassas, VA, USA). Cell cultures were conducted under 37°C in a humidified incubator supplemented with 5% CO_2_. The Dulbecco’s Modified Eagle Medium (DMEM; Invitrogen, Carlsbad, CA, USA) was used to culture UMUC3, RT4, and SV-HUC-1 cells, while the Roswell Park Memorial Institute (RPMI) 1640 Medium was used to culture the T24, 5637, and TCCSUP cells. Ten percent fetal bovine serum (FBS; Invitrogen, Carlsbad, CA, USA) and 1% penicillin-streptomycin were added to the medium.

### RNA isolation and RT-qPCR

The TRIzol reagent (Invitrogen, Thermo Fisher Scientific, Waltham, MA, USA) was used to extract total RNA from BC tissues and above cells, based on the manufacturer’s protocol. One microgram of total RNA, in addition to the PrimeScript RT reagent kit (Takara Bio, Kusatsu, Japan), were utilized to synthesize the complementary DNA (cDNA). RT-qPCR was performed using the SYBR-Green assay (Takara Bio, Kusatsu, Japan) on an ABI 7500 Real-Time PCR system (Applied Biosystems, Waltham, MA, USA).

The U6 snRNA (ID 001973, Thermo Fisher Scientific, Waltham, MA, USA) was used as an internal reference, and the relative miRNA expression was calculated using the 2−ΔΔCq method [17]. The primer sequences (Invitrogen, Thermo Fisher Scientific, Waltham, MA, USA) are listed in Table 1.

**Table 1:** MiRNA and oligonucleotide sequences.

**miRNA**	**Primer sequence (5′ to 3′)**
miR-151a-5p	GCAGGTCGAGGAGCTCACAG
miR-216a-5p	GCAGGTAATCTCAGCTGGCAA
miR-337-3p	GCGTGCTCCTATATGATGCCT
U6	ATGGACTATCATATGCTTACCGAT
miR-337-3p-mimics-F	CUCCUAUAUGAUGCCUUUCUUC
miR-337-3p-mimics-R	AGAAAGGCAUCAUAUAGGAGUU
miR-337-3p-inhibitor	GAAGAAAGGCAUCAUAUAGGAG

### Oligonucleotide transfection

The miRNA mimics, miRNA inhibitor, and negative control (NC) were purchased from Tsingke Biotechnology (Beijing, China). The Lipofectamine 3000 (Invitrogen, Thermo Fisher Scientific, Waltham, MA, USA) was employed to conduct oligonucleotide transfection. UMUC3 and 5637 cells were first seeded in 6-well plates, separately, and grown until they reached 80% confluence. Next, a mixture containing miRNA mimics, NC, miRNA inhibitor, and Lipofectamine 3000 reagent were added to the cells. The sequences of the mimics and inhibitor are listed in Table 1.

### Cell invasion assay

At 48 h post-transfection, the UMUC3 and 5637 cells were harvested and resuspended in serum-free RPMI 1640 medium. After cell counting, 100 μL medium containing 5 × 10^4^ cells were added into the upper chamber of a transwell plate (Corning Incorporated, Corning, NY, USA) that were precoated with Matrigel, while 700 μL medium containing 10% FBS was added to the lower chamber. The cells were incubated for 24 hours under normal conditions. Cells that passed through the transwell membrane were fixed with 4% paraformaldehyde and stained with 0.5% crystal violet dye. The stained cells were imaged and counted under a microscope at ×200 magnification.

### Wound healing assay

A total of 5 × 10^3^ 5637 and UMUC3 cells were seeded in the 6-well plates and were classified into NC, miRNA-inhibitor, and miRNA-mimics groups. 48 h after oligonucleotide transfection, the cells were cultured until they reached 100% confluence. A 200 μl sterile pipette tip was used to scratch the confluent monolayer. The phosphate-buffered-saline (PBS) was used to wash the wounded cell layers three times and the washed cells were incubated with serum-free medium for 48 h. The migration status of wounded cell layers was assessed by imaging the scratched region at 0 and 24 h (magnification ×100).

### Cell proliferation assay

Cell proliferation was assessed via the Cell Counting Kit-8 (CCK-8, Dojindo Molecular Technologies, Rockville, MD, USA) assay. 48 h after transfection, UMUC3 and 5637 cells were seeded (1 × 10^3^ cells/well) into the 96-well plates. After 12, 24, 48, and 72 h of culture, 10 μL of CCK-8 agent was added to each well and incubated for 1 h. The microplate reader (Infinite 200 PRO, TECAN, Männedorf, Switzerland) was used to measure the absorbance at 490 nm.

### Statistical analysis

The GraphPad Prism software (version 8.0) and R software (version 4.0.2) were employed to process all statistical analyses, and statistical significance was set at *p* < 0.05.

### Data availability statement

The original contributions presented in the study are included in the Article/Supplementary Material.

## RESULTS

### Identification of differentially expressed miRNAs

**Figure 1:**
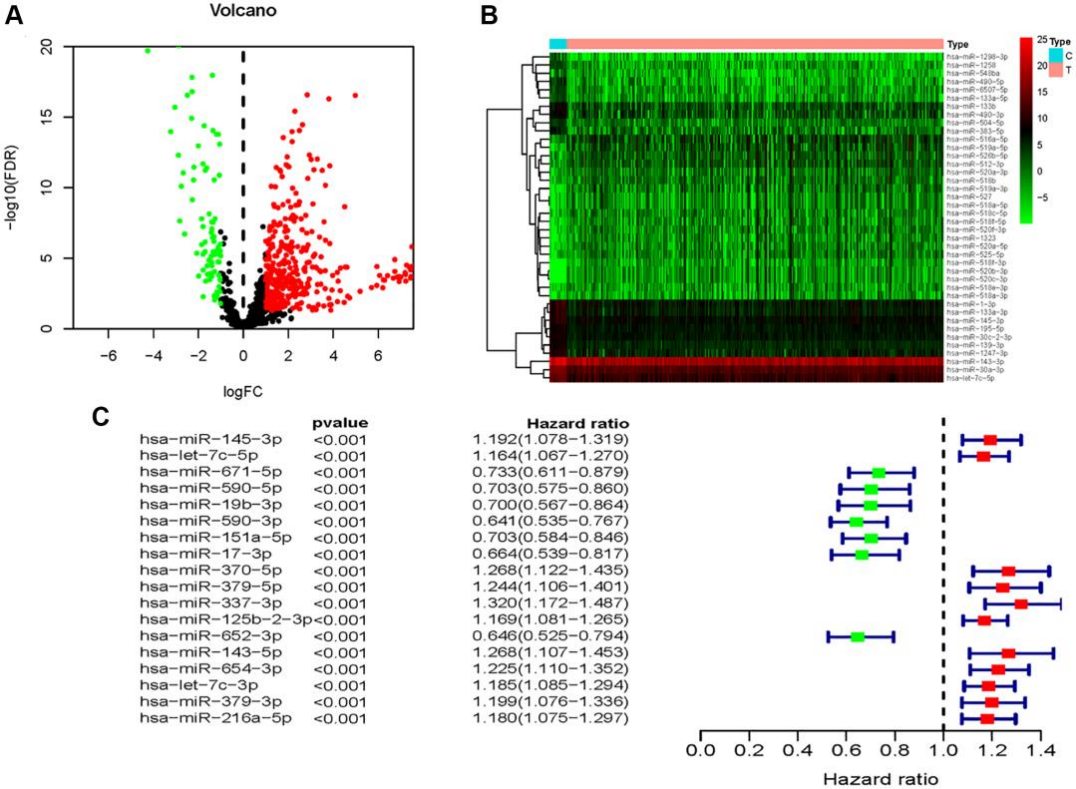
**Identification of OS-related differentially expressed miRNAs between BC and non-tumor tissues.** (**A**) Volcano plot of 437identified differentially expressed miRNAs. (**B**) The heatmap of the unsupervised clustering of the differentially expressed miRNAs. (**C**) The univariate Cox regression analysis for exploration of miRNAs that were significantly correlated with OS of BC patients.

**Figure 2:**
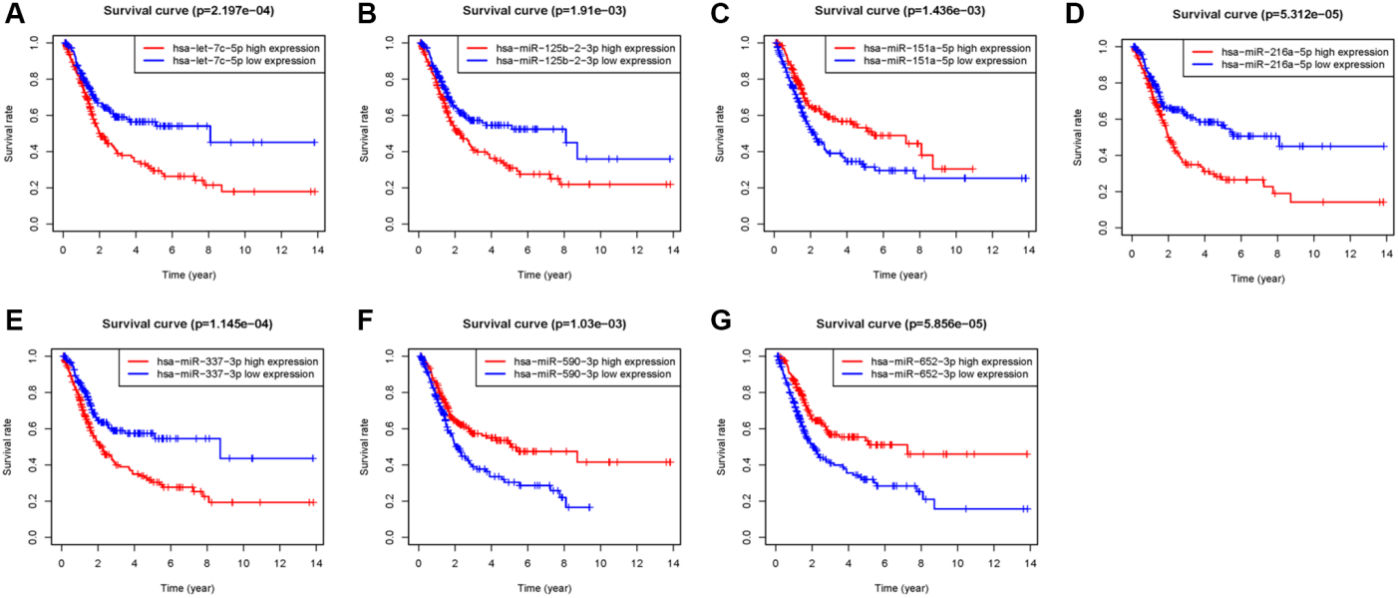
**Kaplan–Meier survival analysis of 7 prognostic-related miRNAs.** (**A**) let-7c-5p, (**B**) miR-125b-2-3p, (**C**) miR-151a-5p, (**D**) miR-216a-5p, (**E**) miR-337-3p, (**F**) miR-590-3p, (**G**) miR-652-3p.

A total of 432 samples comprising, with complete clinicopathological data such as: age, sex, tumor grade, and TNM status, were included in our study. The “limma” package of the R language was utilized to explore the differentially expressed miRNAs between BC samples and non-tumor samples. miRNAs with |log2FC| of >1 and *P* < 0.05, were screened. The volcano plot identified 473 differentially expressed miRNAs (Supplementary Table 1), among which, expression of 379 miRNAs was upregulated and those of 94 miRNAs was downregulated (Figure 1A, Supplementary Table 1). The heatmap demonstrates unsupervised clustering of all the differentially expressed miRNAs (Figure 1B). Univariable Cox regression analysis between the differential expression miRNAs and survival data revealed 18 OS-associated miRNAs (Figure 1C). Multivariate Cox regression analysis filtered 7 miRNAs (miR-151a-5p, miR-216a-5p, miR-337-3p, let-7c-5p, miR-125b-2-3p, miR-590-3p, and miR-652-3p) that may predict prognosis of BC patients (Supplementary Table 2); four were negatively correlated while three were positively correlated to OS (Figure 2A–2G).

### Establishment and evaluation of the predictive 7-miRNA signature

**Figure 3:**
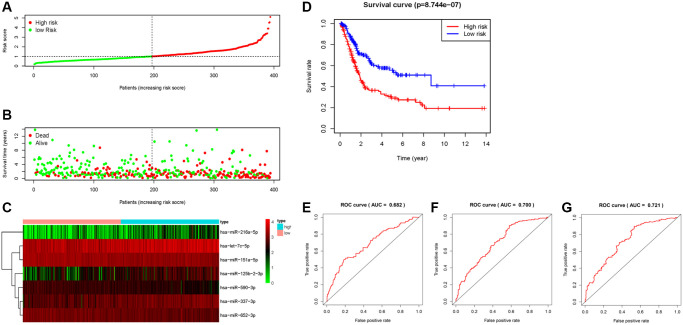
**Construction and evaluation of the predictive 7- miRNA signature.** (**A**) The distribution of the 7-miRNA risk score for each patient. (**B**) Scatter dot plot for survival rates of patients with BC in low- and high-risk groups. (**C**) Heatmap for the different expression of prognostic signature-related miRNAs in different risk groups. (**D**) Kaplan–Meier curve of OS in high- and low-risk groups. (**E**–**G**) ROC curves of the 7-miRNA signature for prediction of 1-, 3- and 5-year OS.

Prognostic miRNAs including: miR-151a-5p, miR-216a-5p, miR-337-3p, hsa-let-7c-5p, miR-125b-2-3p, miR-590-3p, and miR-652-3p were fed into the signature (namely 7-miRNA signature), based on the calculated risk score [risk score = (−0.4762 × let-7c-5p expressions) + (−0.3482 × miR-590-3p expressions) + (−0.2106 × miR-151a-5p expressions) + (0.1619 × miR-337-3p expressions) + (0.3912 × miR-125b-2-3p expressions) + (−0.2371 × miR-652-3p expressions) + (0.1525 × miR-216a-5p expressions)] for each patient. A median value of the risk score was set, and patients were divided into low- and high-risk groups based on this (Figure 3A).

The scatter dot plot indicated that patients in low-risk group presented a longer survival duration (Figure 3B). The expression patterns of these seven miRNAs in the 413 BC patients are displayed in Figure 3C, indicating that high-risk scores tend to express high-risk miRNAs, while low-risk scores were the opposite. Similarly, an increased mortality rate was observed in the high-risk group (Figure 3D). The 7-miRNA signature yielded a satisfactory predictive ability, with 1-, 3-, and 5-years AUC values of 0.682, 0.700, and 0.721, respectively (Figure 3E–3G).

### Prognostic value of the 7-miRNA-signature based on risk score

**Figure 4:**
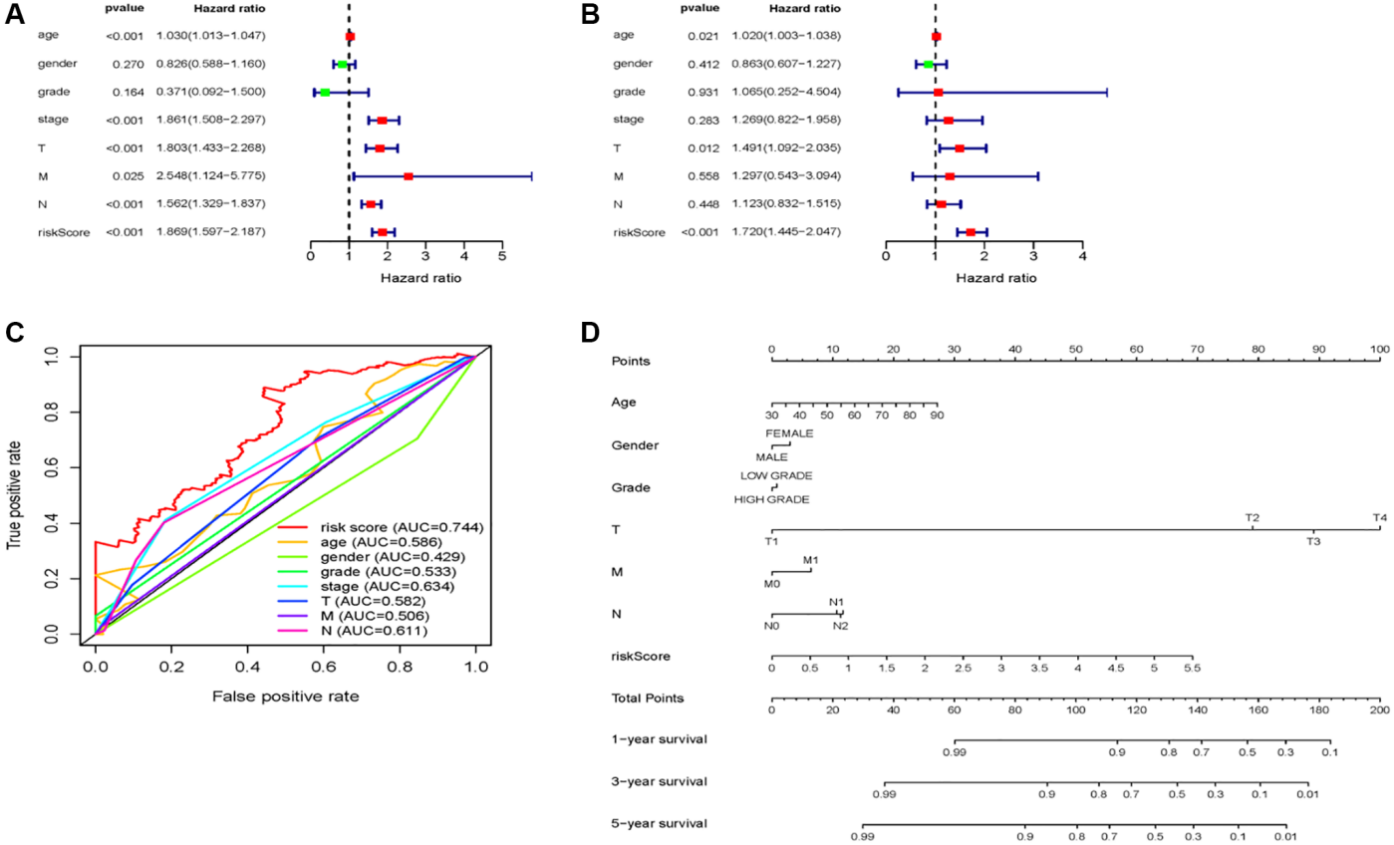
**7 miRNA-signature based risk score predicts OS in patients with BC.** (**A**) Univariable and (**B**) multivariable analyses for the risk score and clinic-pathological features. (**C**) ROC curves of the 7 miRNA-signature based risk score and clinicopathological characteristics. (**D**) A nomogram integrates clinicopathological parameters and the risk score.

The univariate and multivariate Cox analyses were conducted in order to explore the prognostic value of the 7-miRNA-signature based on risk score in BC patients. The risk score of the 7-miRNA signature along with clinicopathological characteristics such as age, sex, grade, stage, and TNM status were used as covariates. Covariates that were significantly associated with survival in univariate Cox analysis (age [*P* < 0.001], stage [*P* < 0.001], T status [*P* < 0.001], N status [*P* < 0.001], M status [*P* = 0.025], and risk score [*P* < 0.001]) were subjected to multivariate Cox analysis. Results indicated that age (HR = 1.020, 95% CI: 1.003–1.038, *P* = 0.021) and risk score (HR = 1.720, 95% CI: 1.445–2.047, *P* < 0.001) are independent prognostic indicators (Figure 4A, 4B). Moreover, multi-parameter ROC curve analyses showed that the risk score achieved the most satisfactory predictive performance compared with clinicopathological characteristics, with an AUC value of 0.744, indicating that the 7-miRNA risk score is a promising prognostic biomarker (Figure 4C).

Next, we created a nomogram that integrating with the 7-miRNA-signature risk score and clinicopathological prognostic factors, which presented better predictive performance for 5-year OS (Figure 4D).

### Target gene prediction and functional enrichment analysis

**Figure 5:**
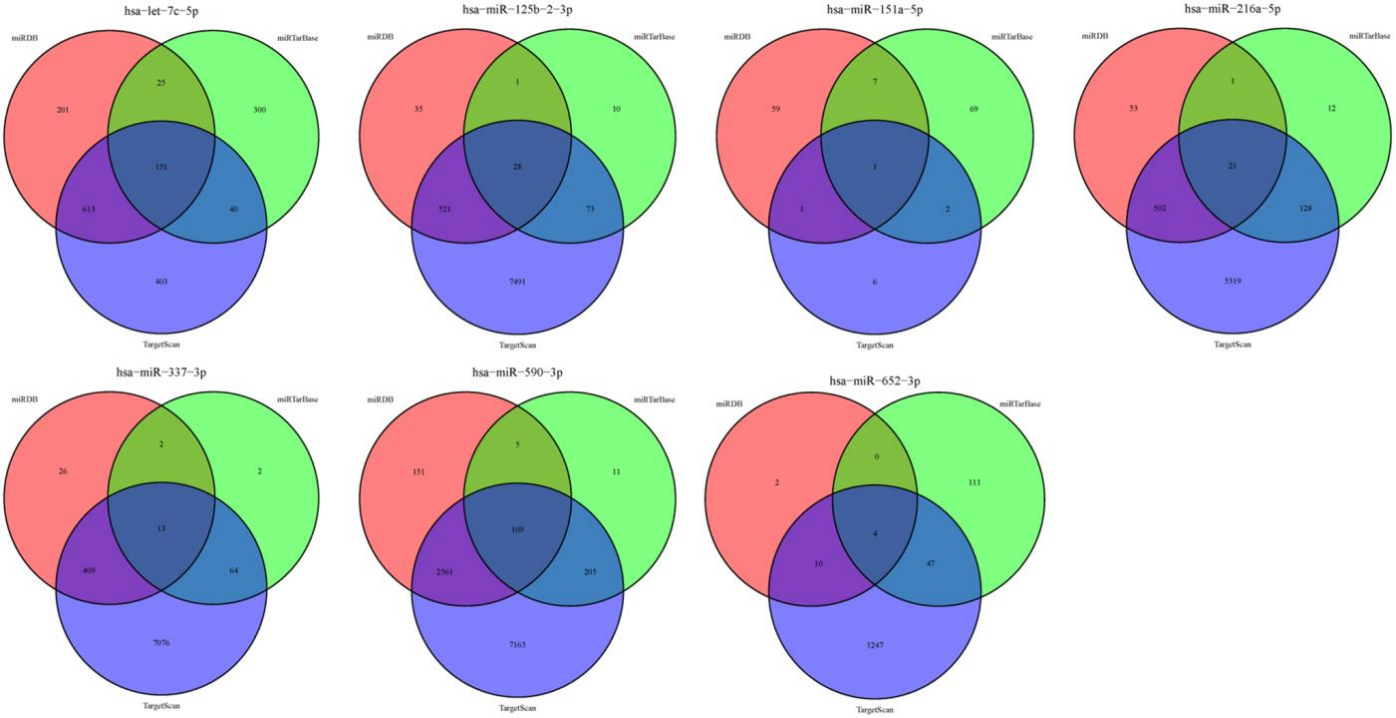
Target genes of the 7 miRNAs.

**Figure 6:**
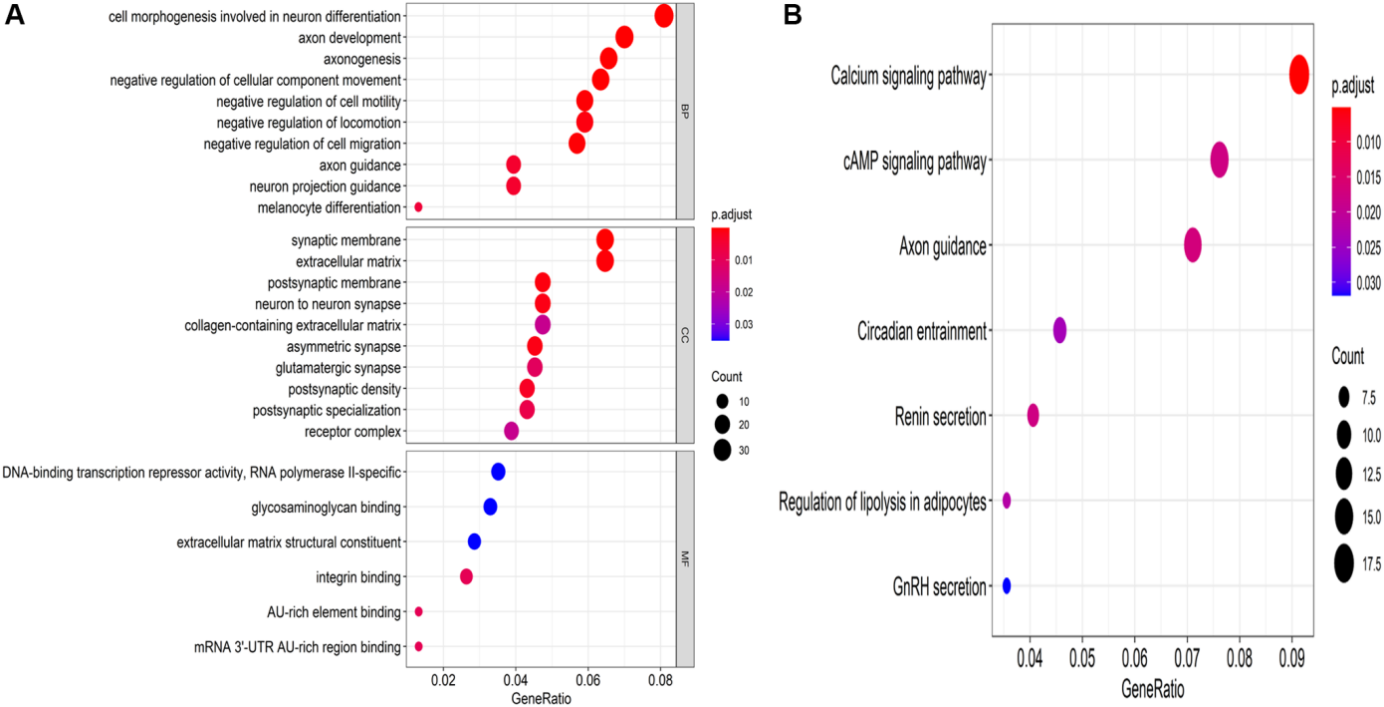
**Underlying molecular functions of the target genes.** (**A**) GO enrichment analysis and (**B**) KEGG pathway enrichment analysis of the target genes.

Overall, 387 overlapping mRNAs were identified via three online analysis tools (Figure 5). GO enrichment analysis indicated that these target genes were mainly enriched in 11 biological processes, 10 cellular components, and 6 molecular functions, among which cell morphogenesis involved in neuron differentiation, axon development, and axonogenesis; synaptic membrane, extracellular matrix, and postsynaptic membrane; and DNA-binding transcription repressor activity, RNA polymerase II-specific, glycosaminoglycan binding, and extracellular matrix structural constituent represented the top three biological processes, respectively (Figure 6A). Moreover, KEGG pathway enrichment analysis was conducted, indicating the following seven pathways were enriched with the target genes: calcium signaling pathway, cAMP signaling pathway, axon guidance, circadian entrainment, renin secretion, regulation of lipolysis in adipocytes, and GnRH secretion (Figure 6B).

### Detection of miR-337-3p, miR-216a-5p, and miR-151a-5p expression in clinical samples and cell lines

**Figure 7:**
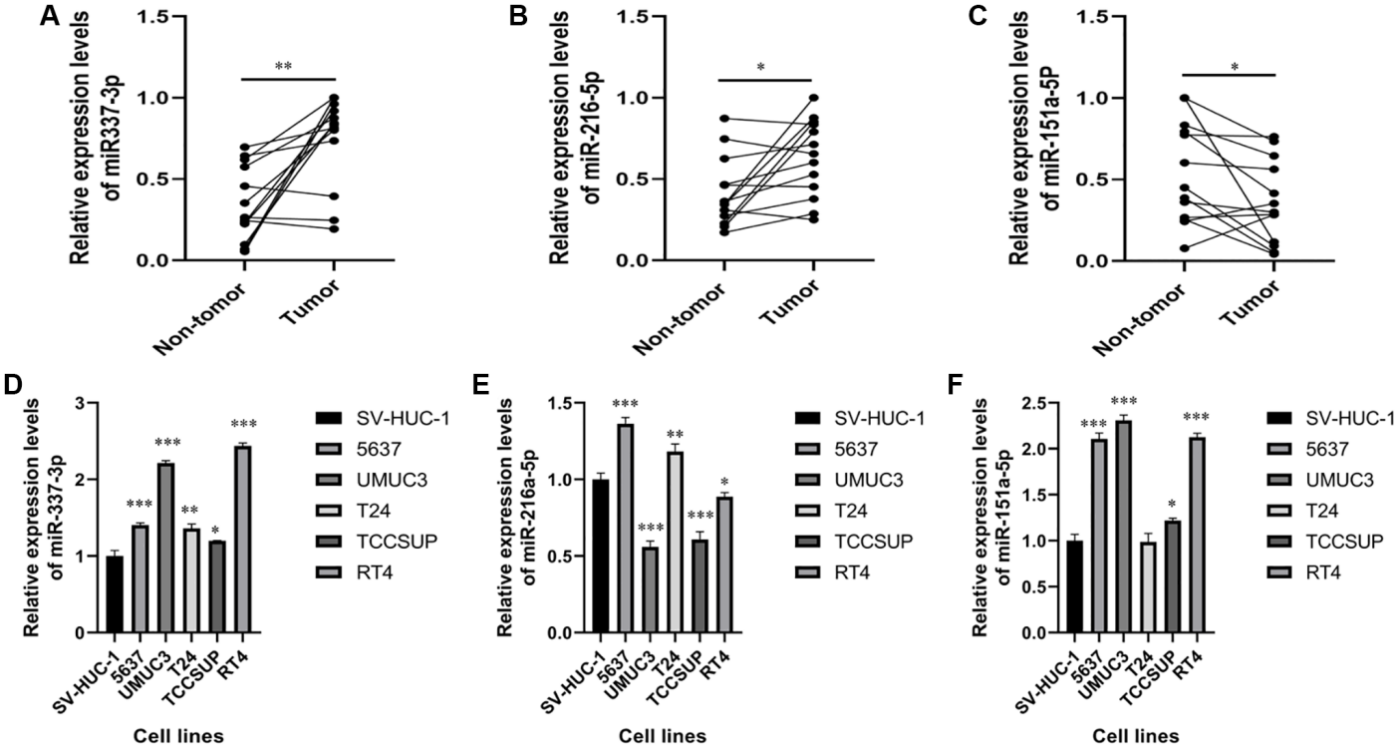
**Expression levels of miR-337-3p, miR-216a-5p and miR-151a-5p expressions in clinical samples and cell lines.** (**A**–**C**) RT-PCR shows that the expression levels of miR-337-3p, miR-216a-5p were significantly higher in BC tissues, while the expression levels of miR-151a-5p were lower in BC tissues, compared with non-tumor tissues. (**D**–**F**) The expression levels of miR-337-3p and miR-151a-5p were increased in the bladder cancer cell lines, while the expression levels of miR-216a-5p was decreased, compared with non-tumorigenic bladder cell line. ^*^p < 0.05, ^**^p < 0.01, ^***^p < 0.001.

MiR-337-3p, miR-216a-5p, and miR-151a-5p expressions were measured in clinical samples and cell lines using RT-qPCR. As shown in Figure 7A–7C, in the BC tissues, the expression levels of miR-337-3p and miR-216a-5p were significantly increased, while the miR-151a-5p expressions were markedly decreased, comparing with non-tumor tissues. Even miR-216a-5p and miR-151a-5p levels were inconsistent with the results obtained in paired clinical samples; miR-337-3p expression was still significantly upregulated in BC cell lines, indicating that miR-337-3p promotes tumorigenesis (Figure 7D–7F). Thus, owing to its consistently high expressions, we speculate that miR-337-3p promotes the development and progression of bladder cancer.

### MiR-337-3p induces proliferation, migration, and invasion in BC cells

**Figure 8:**
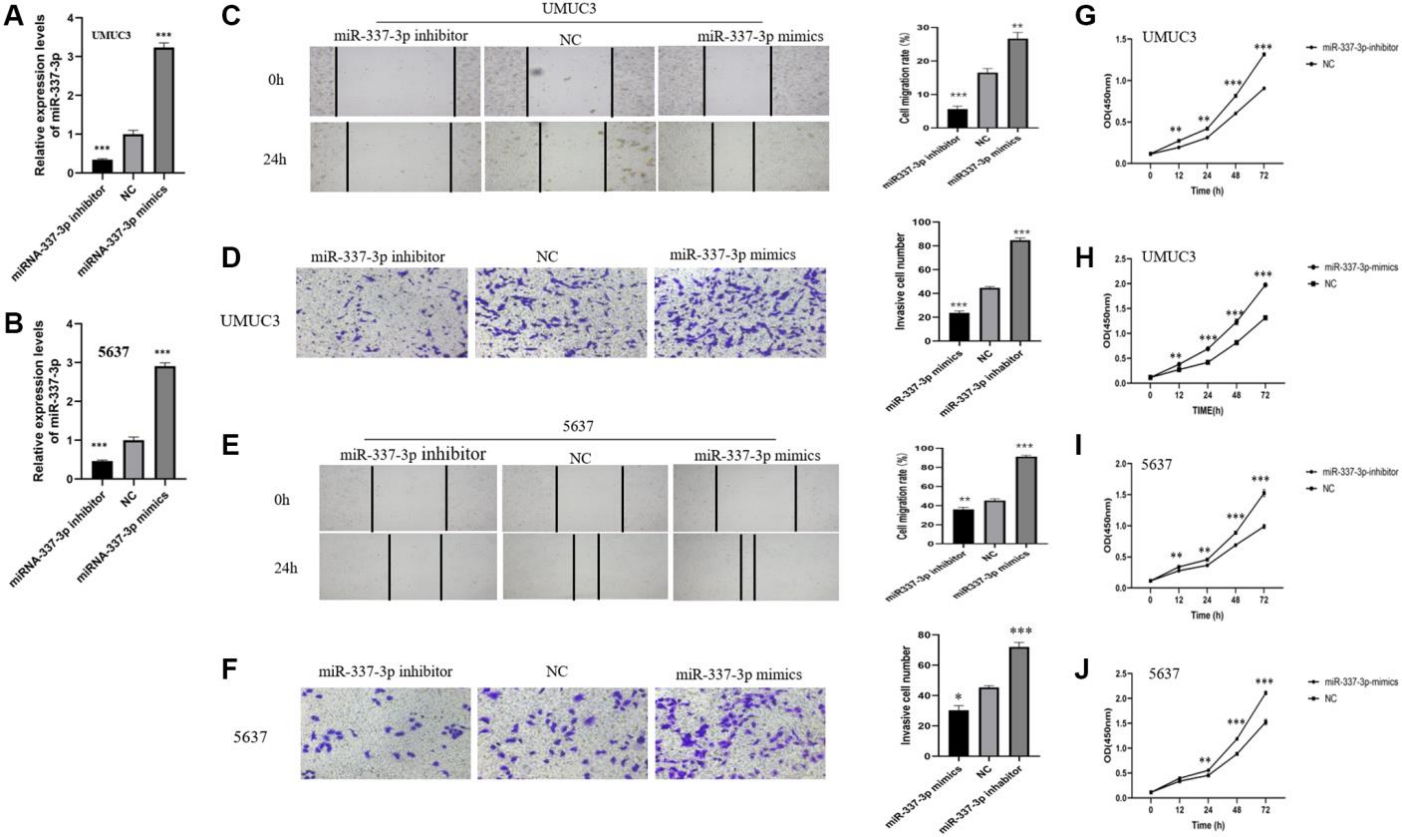
**MiR-337-3p induces proliferation, migration, and invasion in BC cells.** MiR-337-3p expressions in (**A**) UMUC3 and (**B**) 5637 cells were increased by the miR-337-3p mimics transfection and decreased by miR-337-3p inhibitor transfection, compared with the NC group. Wound healing assays (magnification, ×100) and transwell invasion assays (magnification, ×200) revealed that after transfection of miR-337-3p inhibitor, the cell migration rates and invasive cell number of UMUC3 cells (**C**, **D**) and 5637 cells (**E**, **F**) were restrained, while miR-337-3p mimics transfection promoted migration and invasion of UMUC3 (**C**, **D**) and 5637 (**E**, **F**) cells. CCK8 assays indicated that overexpression of miR-337-3p promoted proliferation activities of UMUC3 (**G**, **H**) and 5637 (**I**, **J**) cells, while miR-337-3p inhibitor groups acted the opposite. ^*^p < 0.05, ^**^p < 0.01, ^***^p < 0.001.

The miR-337-3p mimics, NC, and miR-337-3p inhibitor were transfected into UMUC3 and 5,637 cells. The corresponding upregulated or downregulated miR-337-3p expressions were detected (Figure 8A, 8B). The wound healing assay showed that miR-337-3p overexpression obviously increased the migration rate of UMUC3 and 5637 cells, while opposite results were observed in miR-337-3p inhibitor groups (Figure 8C, 8E). Transwell invasion assays revealed that the group overexpressing miR-337-3p showed increased cell invasion, while the groups transfected with miR-337-3p inhibitor did not (Figure 8D, 8F). In addition, as demonstrated in Figure 8G–8J, the CCK-8 assay reduced cell proliferation in UMUC3- and 5637- cells transfected with miR337-3p inhibitor, while cells transfected with miR-337-3p mimics showed increased cell proliferation. In conclusion, overexpression of miR-337-3p enhances the proliferation, migration, and invasion abilities of BC cells.

## DISCUSSION

Traditional predictive indicators, such as TNM staging and pathological grade, have a limited ability to predict BC prognosis. Considering the high mortality rate of patients with NMIBC, novel prognostic indicators that can more accurately predict patient prognosis are urgently needed. In the present study, seven BC prognosis-related miRNAs (miR-151a-5p, miR-216a-5p, miR-337-3p, hsa-let-7c-5p, miR-125b-2-3p, miR-590-3p, and miR-652-3p) were identified to establish a 7-miRNA signature, which obtained an AUC value of 0.721 in terms of predicting 5-year OS. The risk score of the 7-miRNA signature provided an increased predictive ability (AUC = 0.744). The expression levels of 3 miRNAs (miR-151a-5p, miR-216a-5p, and miR-337-3p) along with their potential molecular regulation in BC cells were investigated via experiments.

Several previous studies have reported the predictive value of miRNA signatures in patients with BC. Yin et al. [12] developed a 21-miRNA signature to predict the OS of BC patients, proving that bioinformatic-rooted miRNA-signatures can be used for predicting prognosis in BC patients. Using meta-analysis, Zhou et al. [18] screened eight miRNAs that correlate with the survival of patients with BC, and built an 8-miRNA signature with predictive and prognostic value. Their predictive miRNA-signatures achieved approximate AUCs of 0.7, but neither Yin et al. nor Zhou et al. validated their findings *in vivo* or *in vitro* experiments or revealed the underlying molecular mechanisms. In this study, 473 differentially expressed miRNAs were observed between 413 BC specimens and 19 non-tumor specimens using the “limma” package in R. The univariate and multivariate Cox regression analysis yielded seven novel prognosis-related miRNAs, which were employed to build a 7-miRNA signature. The signature-based risk score was calculated, which indicated that patients in the low-risk group had a longer OS compared with high-risk group. The 7-miRNA signature and risk score exhibited favorable predictive abilities, with AUC values of 0.721 and 0.744, respectively. In addition, to facilitate clinical application, we constructed a nomogram that incorporated risk scores and clinicopathological factors, showing a better prognostic value than a single parameter.

To further explore the molecular functions of these miRNAs, we attempted to validate their expressions in paired clinical samples and cell lines. A literature review of these seven miRNAs, using studies published on PubMed, revealed that these miRNAs are involved in the progression of bladder cancer or other cancers. Let-7c-5p is considered to be associated with the pathological grade of colon cancer, and may restrain lymph node metastasis in patients with breast cancer [19, 20]. Overexpression of miR-125b-2-3p may promote metastasis of clear cell renal cell cancer by targeting EGR1, leading to poor prognosis [21]; it also plays an important role in regulating the proliferation of colorectal cancer and oral squamous cell carcinoma by acting as a downstream target of long noncoding RNAs, XIST and AC007271.3 [22, 23]. MiR-590-3p reportedly promotes nasopharyngeal and ovarian cancer carcinogenesis, and acts to inhibit invasion and metastasis in triple-negative breast cancer [24–26]. Ji et al. [27] demonstrated that miR-652-3p may be a serum indicator to predict tumor relapse and treatment outcomes in patients with colorectal cancer. In addition, hsa-let-7c-5p, miR-125b-2-3p, miR-590-3p, and miR-652-3p have been reported to regulate biological functions such as migration, proliferation, invasion, and apoptosis of BC [28–31]. Although miR-151a-5p is reportedly a prognostic factor for prostate cancer and lung adenocarcinoma [32, 33], miR-216a-5p act acts a suppressor in pancreatic cancer and breast cancer by targeting downstream mRNAs [34, 35]. Overexpression of miR-337-3p suppresses the migration and invasion of cervical cancer and ovarian cancer cells [36, 37]. These studies indicate that the miRNAs used to construct the 7-miRNA signature in our study may be clinically important cancer biomarkers involved in multiple signaling pathways in various tumors. However, to the best of our knowledge, miR-151a-5p, miR-216a-5p, and miR-337-3p have not been verified to predict prognosis or regulate molecular functions of BC. Therefore, we investigated the expression of miR-151a-5p, miR-216a-5p, and miR-337-3p in clinical samples and cell lines. RT-qPCR results show that compared with non-tumor tissues and non-tumorigenic bladder cell lines, miR-337-3p is overexpressed in tumor tissues and BC cell lines, while inconsistent results were observed in miR-151a-5p and miR-216a-5p expressions. Considering that patients with decreased miR-337-3p expression had a longer survival time (Figure 2), miR-337-3p was preliminarily identified to affect the biological progression of BC cells.

To better understand the molecular functions of these seven miRNAs, their target genes were identified using bioinformatic tools. We conducted GO enrichment and KEGG pathway enrichment analyses. GO terms showed that these target genes mainly affect biological functions, such as cell morphogenesis involved in neuron differentiation and axon development. KEGG pathway enrichment analysis revealed that these target genes are highly enhanced in the calcium signaling pathway, cAMP signaling pathway, axon guidance, and so on. Based on these results, we examined the molecular functions of miR-337-3p via wound healing, transwell invasion, and CCK8 assays, demonstrating that forced expression of miR-337-3p enhances the proliferation, migration, and invasion abilities of BC cells.

This study had several limitations. First, as a retrospective study, selection bias may have been inevitable. Second, we did not divide the dataset into training and validation sets due to the insufficient sample size. Thus, large-scale prospective studies and additional *in vivo* and *in vitro* validation studies are warranted to confirm our findings and reveal the molecular mechanisms underlying the effects of miR-337-3p action in BC.

## CONCLUSIONS

In this study, we present a novel 7-miRNA signature that may be a promising prognostic biomarker for patients with BC. This 7-miRNA signature can act as an independent prognostic indicator for BC. Integrating the 7-miRNA signature and other prognostic factors, we established a predictive model that showed a better prognostic value than a single parameter. We further explored the potential correlations between miR-151a-5p, miR-216a-5p, and miR-337-3p and the malignant biological behavior of BC cells, and found that miR-337-3p may not only be a novel prognostic biomarker for patients with BC, but also acts as a candidate therapeutic target in BC treatment.

## Supplementary Materials

Supplementary Table 1

Supplementary Table 2
