# Supplementary material for: Development of a 7-miRNA prognostic signature for patients with bladder cancer
Source: Aging (Albany NY). 2022 Dec 21;14(24):10093–106. doi: 10.18632/aging.204447 (PMC9831742; doi:10.18632/aging.204447)
Supplement: Supplementary Table 2 [file aging-14-204447-s002.pdf]

SUPPLEMENTARY TABLE

Supplementary Table 2. Multivariate Cox regression analysis of the 7 candidate miRNAs.

ID	coef	HR	HR.95L	HR.95H	*p*-value
hsa-let-7c-5p	−0.476175	0.621155	0.464975	0.829793	0.00127
hsa-miR-590-3p	−0.348215	0.705947	0.580388	0.85867	0.000492
hsa-miR-151a-5p	−0.210616	0.810085	0.644008	1.018989	0.071977
hsa-miR-337-3p	0.1619093	1.175754	1.006487	1.373487	0.041202
hsa-miR-125b-2-3p	0.3912236	1.478789	1.139598	1.918938	0.003251
hsa-miR-652-3p	−0.237091	0.788919	0.616345	1.009813	0.059778
hsa-miR-216a-5p	0.1524685	1.164706	1.038267	1.306541	0.00931
